# Identification and Comprehensive Analysis of *OFP* Genes for Fruit Shape Influence in Mango

**DOI:** 10.3390/genes15070823

**Published:** 2024-06-21

**Authors:** Qiuping Wu, Rui Xia, Jie Yang, Rong Chen, Zaohai Zeng, Chao Fan

**Affiliations:** 1 Institute of Fruit Tree Research, Guangdong Academy of Agricultural Sciences, Key Laboratory of South Subtropical Fruit Biology and Genetic Resource Utilization, Ministry of Agriculture and Rural Affairs, Guangdong Provincial Key Laboratory of Tropical and Subtropical Fruit Tree Research, Guangzhou 510642, China; wqp205947@163.com (Q.W.);; 2 Guangdong Litchi Engineering Research Center, College of Horticulture, South China Agricultural University, Guangzhou 510642, China; rxia@scau.edu.cn

**Keywords:** mango (*Mangifera indica* L.), OVATE family proteins, expression pattern, fruit development

## Abstract

OVATE family proteins (OFPs) are a class of plant-specific proteins with a conserved OVATE domain that play fundamental roles in fruit development and plant growth. Mango (*Mangifera indica* L.) is an economically important subtropical fruit tree characterized by a diverse array of fruit shapes and sizes. Despite extensive research on OFPs across various species, there remains a scarcity of information regarding OFPs in mango. Here, we have successfully identified 25 OFP genes (*MiOFP*s) in mango, each of which exhibits the conserved OVATE domains. The *MiOFP* gene exhibit a range of 2–6 motifs, with all genes containing both motif 1 and motif 2. Phylogenetic analysis on 97 OFPs (including 18 AtOFPs, 24 SlOFPs, 25 MiOFPs, and 30 OsOFPs) indicated that MiOFPs could be divided into three main clades: clade I, II, and III. Comparative morphological analysis identified significant variations in fruit longitudinal diameter, fruit transverse diameter, and fruit shape index between two distinct shaped mango cultivars (‘Hongxiangya’ and ‘Jingpingmang’) at DAP5, DAP7, and DAP10 stages. The subsequent examination of paraffin sections revealed distinct patterns of cell elongation. The majority of *MiOFP* genes exhibited predominantly expressed in developing organs, specifically flowers and immature fruits, while displaying distinct expression patterns. RNA-Seq analysis revealed significant disparities in the expression levels of several *OFP* genes, including *MiOFP*5, *MiOFP*11, *MiOFP*21, *MiOFP*22, *MiOFP*23, and *MiOFP*25, between the two mango cultivars. These findings suggest that these six genes may play a crucial role for fruit shape in mango, especially the *MiOFP*22. The findings of this study have established a basis for future investigations into *MiOFP*s in mango, offering a solid foundation for further research in this field.

## 1. Introduction

Mango (*M*. *indica* L.) is an economically important species whose fruit is regarded as delicious and nutritious. Globally, there are about 26,000 mango accessions including landraces, breeding lines, advanced cultivars, and wild species held in various field gene banks in Australia, Indonesia, Sierra Leone, India, Thailand, United States of America, and other countries [1]. These cultivated mangoes exhibit a diverse range of shapes, such as round, S-shaped, oval, ivory-shaped, rectangular, elongated, and more [2]. Consequently, studying the mechanism behind mango fruit shape formation is advantageous in meeting consumer demands and production processing requirements.

The shape of a fruit is a key trait in horticulture that plays a significant role in determining the commercial value of fruits. Not only does it accommodate various methods of consumption and to meet consumers’ preferences, but it also affects the harvesting methods of fruits. For instance, elongated and blocky tomatoes are preferred for processing purposes, while round and visually appealing fruits are ideal for the fresh market and slicing applications [3,4]. Similarly, regular mango varieties such as round are easier to manage postharvest than other irregular fruit shapes such as ivory-shaped. However, the underlying factors that contribute to the development of diverse fruit shapes and the production system is poorly investigated [5,6].

In recent years, the molecular genetic mechanisms of fruit shape regulation has been preliminarily investigated. Numerous genes and regulatory factors have been identified and characterized for their involvement in this process. Among them, the *OFP* gene family receives significant attention [7,8,9]. OFP proteins have been found to play pivotal roles in the regulation of various aspects of plant growth and development. These encompass ovule development, vascular development, fruit shape, and other processes [10,11,12]. In Arabidopsis, *AtOFP*5 is believed to play a role in the early developmental stages of the embryo sac and is essential for female gametophyte development [13]. In rice, it has been confirmed that *OsOFP*2 is associated with lignin biosynthesis and regulates vascular development [14]. Additionally, *OsOFP*19 negatively modulates the response to brassinosteroids (BR) and integrates it with the pattern of cell division to influence plant architecture, including grain shape [15]. In tomato, the OVATE (SlOFP1) and SlOFP20 proteins also interact with the TONNEAU1-recruiting motif family of proteins (TRMs) to regulate multicellular growth in plants and subsequently influence fruit shape [16,17]. Studies have already found that the OFP assumes a crucial role in regulating plant growth and fruit shape [18,19,20]. Numerous OFP proteins have been identified in different fruits, including the *VvOFP*s in grape [21], *ZjOFP*s in Chinese Jujube [22], and *MdOFP*s in apple [23], highlighting the essential functions of OFP proteins in various plant species. However, the exploration of OFP proteins in mango remains limited.

In this study, bioinformatic analyses including those involving sequence similarities, gene structures, motif compositions, gene duplications, and chromosome distribution, were conducted. The phylogenetic analysis of 97 OFPs, which included 18 AtOFPs, 24 SlOFPs, 25 MiOFPs, and 30 OsOFPs, revealed that the 25 MiOFPs could be classified into three primary clades: clades I, II, and III. Furthermore, the study discussed the morphological and cellular changes observed during mango fruit development in different fruit varieties, along with the expression variations of MiOFP family members throughout fruit development. The result provides a foundation for the identification and further functional analysis of MiOFPs family members in mango.

## 2. Materials and Methods

### 2.1. Plant Materials

In this study, two mango cultivars (‘Hongxiangya’ and ‘Jingpingmang’), were selected as plant materials. ‘HXY’ and ‘JPM’ are two mango varieties that display remarkable difference in fruit shape. HXY exhibits an elongated shape, exemplifying a high fruit shape index ranging from 1.89 to 3.04, whereas ‘JPM’, characterized by a round fruit shape, showcases a fruit shape index spanning from 0.89 to 1.11 after maturity. These cultivars were obtained from the Institute of Fruit Tree Research in Chaozhou, Guangdong, China, and were cultivated in a greenhouse under standardized growth conditions. After flower bud differentiation, different tissue samples were collected, including flower buds, leaves, stems, and fruits. Fruit samples were collected at three different stages of fruit development, specifically at 5, 7, and 10 days after pollination, which were referred to as DAP5, DAP7, and DAP10, respectively.

### 2.2. Identification, Characterization, and Phylogenetic Analyses of MiOFPs

The GFF sequence file for the mango genome ‘Alphonso’ and the HMM file for the Ovate domain (PF04844) were acquired from Mangobase (https://mangobase.org/ (accessed on 20 December 2023)) and Pfam database (http://pfam.xfam.org/ (accessed on 20 December 2023)), respectively [24]. Subsequently, the local mango protein database was searched using HMMER3 v3.4 (http://hmmer.janelia.org/ (accessed on 20 December 2023)), resulting in the identification of 26 candidate gene family members. *Arabidopsis thaliana* OFP protein sequences were employed as query sequences and subjected to blastp against the mango protein database using TBtools-Ⅱ [25], with E-values set to 1 × 10^−5^. Eventually, 25 members of the *MiOFP* gene family were identified and further screened to remove any duplicates.

The conserved domain of the OFP protein was predicted using NCBI CD-search (https://www.ncbi.nlm.nih.gov/Structure/cdd (accessed on 22 December 2023)) and Pfam (http://pfam.xfam.org/ (accessed on 22 December 2023)). Furthermore, the conserved motifs were predicted using MEME (http://meme.nbcr.net/meme4-1/cgi-bin/meme.cgi (accessed on 22 December 2023)). The upstream 2 kb sequences relative to the start codon of *MiOFP* genes were obtained from the mango genome database. The cis-acting regulatory elements in these regions were identified using the PlantCARE database (http://bioinformatics.psb.ugent.be/webtools/plantcare/html/ (accessed on 22 December 2023)) and visualized with TBtools Ⅱ [25]. 

The sequences of OFP proteins from *M*. *indica*, *A*. *thaliana*, and *Solanum lycopersicum* were downloaded from the GenBank database. These sequences were aligned using MAFFT v7.490 [26]. *Oryza sativa*, *A*. *thaliana*, and *S*. *lycopersicum* OFP sequences were obtained from the NCBI (https://www.ncbi.nlm.nih.gov/genome/ (accessed on 25 December 2023)). A maximum likelihood (ML) tree was constructed using IQ-TREE v.1.6.12 [27]. The best-fitting nucleotide substitution model, TPM2u+F+G4, was determined using the Akaike Information Criterion (AIC) by ModelFinder [28] in the IQ-TREE package and 1000 bootstrap replicates.

### 2.3. Phenotypic and Histological Observations

Fruit length and diameter of ‘HXY’ and ‘JPM’ mango fruits were measured at 1, 3, 5, 7, 10, 17, 24 and 31 DAP (days after pollination). To observe the changes of cells in the developed fruit, 0.5-cm-thick samples were cut along longitudinal axes from the mesocarp in the middle of each fruit at DAP5, DAP7, and DAP10. Sliced samples were immediately fixed in the formaldehyde–acetic acid–ethanol Fixative (FAA: 70% ethyl alcohol: 10% formaldehyde: 5% glacial acetic acid = 10:2:1). Subsequently, 4 µm thick microtome sections were prepared and stained with hematoxylin–eosin staining (HE) [29]. After staining, the tissues were observed under a microscope (Nikon, E200, Tokyo, Japan) with micrographs captured by an HQ Image C630 digital camera.

### 2.4. RNA-Seq Analysis

Samples were collected at three different stages of fruit development, specifically at 5, 7, and 10 days after pollination, named DAP5, DAP7, and DAP10, respectively. Immediately after collection, the samples were flash-frozen and pulverized using a mortar and pestle. Total RNAs were extracted from fruit pulp using the RNAprep Pure Plant Kit (TIANGEN, Beijing, China). RNA-seq libraries were sequenced on an Illumina NovaSeq 6000 instrument (BMK, Beijing, China) with 150-bp paired-end reads. Approximately 6G of raw data were generated for each sample. The raw reads underwent quality control and adapter sequence removal using fastp [30]. The clean data were aligned to the reference genome of mango cultivar A ‘lphonso’ [24] using the STAR 2.7.11b (https://github.com/alexdobin/STAR (accessed on 10 June 2024)). Transcripts were assembled and merged using StringTie v1.3.3b (http://ccb.jhu.edu/software/stringtie/ (accessed on 10 June 2024)). The gene expression level was determined according to the transcripts per kilobase of exon model per million mapped (TPM) method [31] and gene expression profiles were visualized by heatmap via TBtools-II [25]. The DESeq2 Bioconductor package was utilized to identify differentially expressed genes (DEGs: log_2_FoldChange > 2 and padj < 0.05 or log_2_FoldChange < −2 and padj < 0.05) between the two cultivars at each ripening stage.

### 2.5. Total RNA Extraction and RT-qPCR Analysis

Total RNA of 18 samples was extracted by column plant RNAout kit (Tiandz, Beijing, China) according to the manufacturer instructions. First-strand cDNA was synthesized from 1 μg total RNA using the GoScript Reverse Transcriptase (Promega, A5003). Quantitative reverse transcription polymerase chain reaction (RT-qPCR) was performed using THUNDERBIRD qPCR MIX QPS-201 (Toyobo, Shanghai, China) on a LightCycler 480 Thermal Cycler (Roche, Basel, Switzerland). *MiActin* was used as an internal control for sample normalization during real-time RT-PCR analysis (2^−ΔΔCt^ method) [32,33]. The RT-qPCR primers were designed in Primer3 2.3.7 and listed in Table 1. Each expression profile was independently verified in three biological replicates. 

## 3. Results

### 3.1. Identification of OFP Genes in Mango

**Figure 1:**
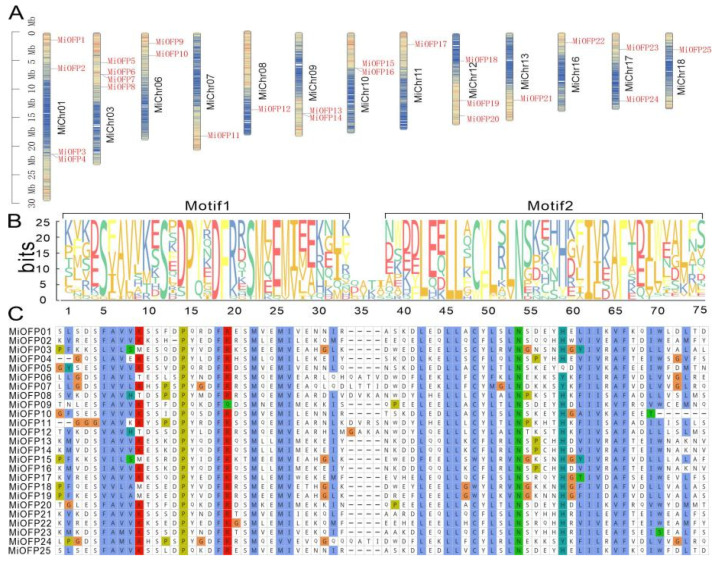
Locations, multiple-sequence alignment of *MiOFP* in mango: (**A**) Chromosomal locations of mango *MiOFP* genes. The intensity of the color of the chromosome is directly proportional to the gene density in that particular region; high gene density: red; low gene density: blue; (**B**) Two conserved motifs in OVATE domain. The overall height of each stack represents the conservation of the sequence at that position, and the height of the letters within each stack indicates the relative frequency of the respective amino acid; (**C**) The OVATE domain sequence alignment of mango *OFP*s. Identical or similar amino acids were shaded in same color box.

The sequences of 19 AtOFPs were used as queries for BLASTp searches to identify *OFP* genes from the genome of “Alphonso” mango. In total, 25 *OFP* genes were identified and designated as *MiOFP*1–*MiOFP*25, based on their respective chromosomal locations (Figure 1A and Table 2). The length of the MiOFP proteins ranged from 178 amino acids (MiOFP16) to 411 amino acids (MiOFP9). The relative molecular mass of these proteins varied from 20,108.71 Da (MiOFP16) to 47,439.15 Da (MiOFP9). Furthermore, their isoelectric points (PIs) ranged from 4.62 (MiOFP18) to 9.71 (MiOFP10). Notably, all of the OFP proteins exhibited negative hydrophobicity values, indicating that these proteins have hydropathicity. For subcellular localization, 17 out of the 25 MiOFPs (68%) were identified to localize in the chloroplast, while only 6 out of the 25 (24%) were in the nucleus (Table 2).

### 3.2. Sequence Alignment, Structure, and Phylogenetic Analysis of the MiOFPs

To better illustrate the gene structure of *MiOFP*s, the OVATE domains were displayed by aligning the conserved OVATE regions in mango. To further investigate whether the OVATE domain of MiOFPs are highly conserved, this study aligned 25 MiOFPs in mango by MAFFT v7.490 [26] and identified the conserved motifs via MEME v5.0 (http://meme-suite.org/ (accessed on 28 January 2024)). The results showed that the OVATE domain contained two conserved motifs, in which they shared some conserved amino acid residues (Figure 1B,C). For example, the S12, P15, D18, F19, M23, L45, L46, N53, I61, and F65 amino acid residues in the OVATE domain were conserved in all MiOFPs.

**Figure 2:**
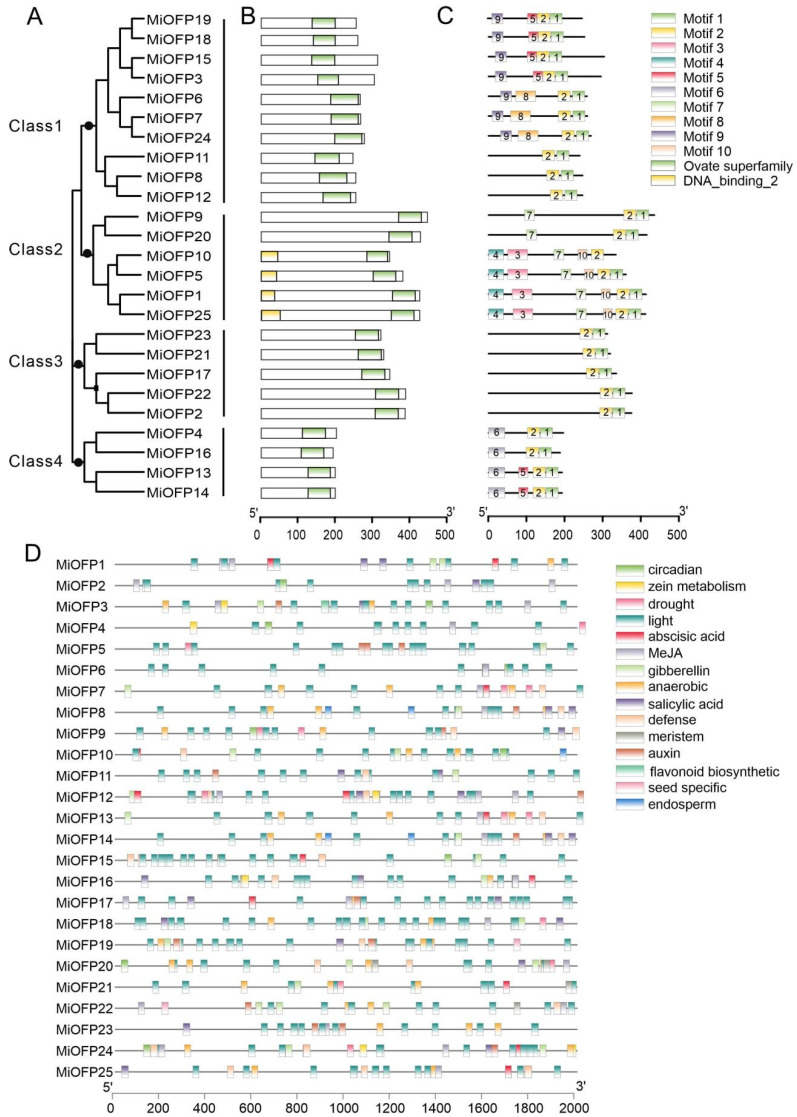
Phylogenetic relationship, conserved domains, conserved motif and promoter analyses of MiOFPs: (**A**) Phylogenetic relationship among the mango OFP proteins. Four clusters are labeled as Class 1, Class 2, Class 3, and Class 4; (**B**) Conserved domains of MiOFPs; (**C**) Conserved motifs of MiOFPs; (**D**) Analysis of cis-acting elements in the promoter region of the *MiOFP* gene family.

Following this, an analysis of the phylogenetic relationship and conserved domains of MiOFPs revealed that they can be categorized into four distinct classes, all of which exhibit the presence of the ovate superfamily domain. Furthermore, MiOFP1, MiOFP5, MiOFP10, and MiOFP25 were additionally identified to possess the DNA binding 2 domain, as depicted in Figure (Figure 2A,B). Conserved motif analyses showed all *MiOFP* genes have motif 1 and 2 (Figure 2C). 

The analysis of promoter regions has shown that these *MiOFP* genes bear cis-elements that associate with plant hormone responses, plant growth and development, as well as responses to biotic and abiotic stresses. Hormone response elements encompass various hormones such as abscisic acid, auxin, gibberellin, MeJA, and salicylic acid. Growth and development-related elements consist of meristem-related elements, seed-specific elements, and endosperm expression-related elements. Abiotic stress response elements predominantly involve responses to light and drought. Furthermore, there are also elements associated with circadian responses (Figure 2D).

**Figure 3:**
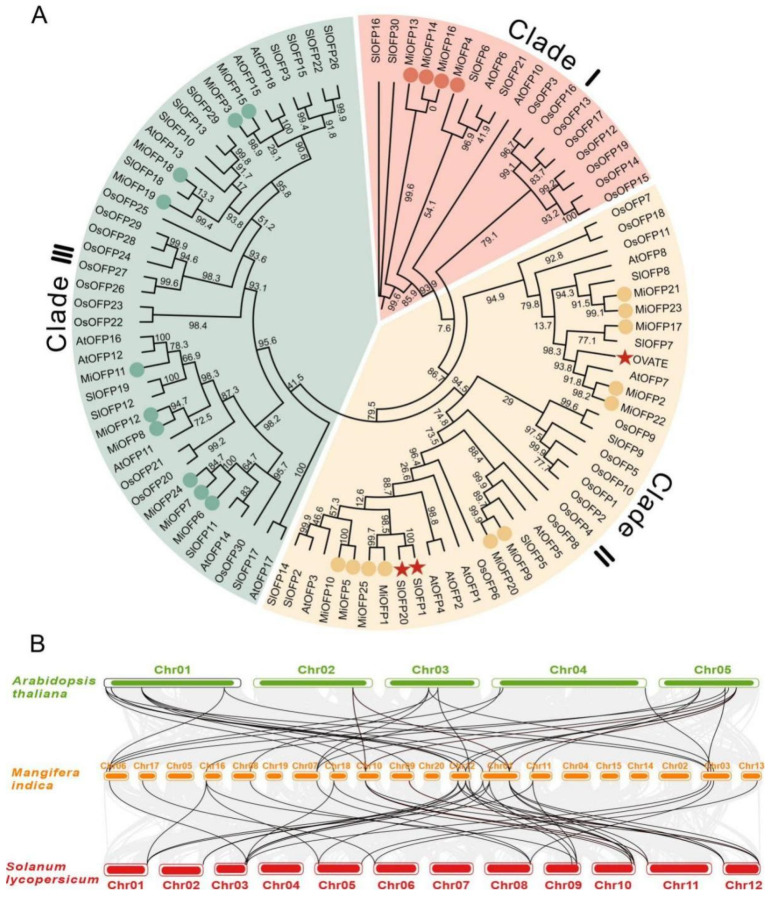
Phylogenetic and collinearity analysis of OFPs: (**A**) Numbers on branches indicate bootstrap values carried out with 1000 bootstrap replicates. Red star, OVATE, SlOFP1, and SlOFP20 of tomato (*S*. *lycopersicum*); circle, mango (*M*. *indica*). AtOFP, *A*. *thaliana* OFPs; SlOFP, tomato OFPs; OsOFP, rice OFPs; and MiOFP, mango OFPs; (**B**) Genome collinearity analysis was performed in *A. thaliana*, *M. indica*, and *Solanum lycoperaicum*. Black line represent the conserved OFP pairs between genomes.

To further understand the evolutionary relationships of the mango OFP proteins, a phylogenetic tree was generated based on the sequence alignments of 25 full-length OVATE domain-containing proteins and 18 *A*. *thaliana* OFPs (*AtOFP*s), 31 *S*. *lycopersicum* OFPs (*SlOFP*s), and 30 *Oryza sativa* (*OsOFP*s). These 97 *OFP*s were classified into three main groups: Clade I, Clade II, and Clade III, comprising 18, 38, and 41 members, respectively (Figure 3A). Among them, *MiOFP*21, *MiOFP*23, *MiOFP*17, *MiOFP*2, and *MiOFP*22, are positioned on the same subbranch of Clade II. This suggests a noteworthy sequence similarity among them. Evolutionary studies on the *OFP* gene family also suggest that it had already formed before the divergence of monocotyledons and dicotyledons, and the number of *OFP* members is greater in monocotyledonous plants than in dicotyledonous plants, indicating that the *OFP* gene family exhibits both functional conservation and specificity during the evolution process. The genomes of Arabidopsis and tomato contain 18 and 26 *OFP* gene family members, respectively, while mango harbors 25 *OFP* family members. Comparative synteny analysis demonstrated a relatively stable number of OFP family members across these species, suggesting the preservation of the gene family size without noticeable expansion or contraction (Figure 3B).

### 3.3. Expression Analysis of MiOFP Genes in Different Tissues

**Figure 4:**
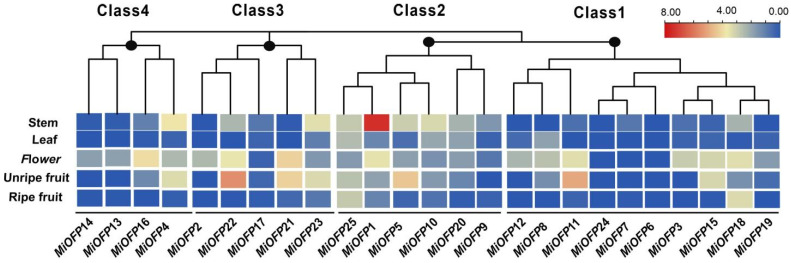
Heatmap of mango MiOFPs gene expression in four tissues (five samples). The color key represents the relative transcript abundance of the MiOFP genes mango tissues.

To explore the role of these *MiOFP* genes, transcriptome analysis was carried out to investigate the expression patterns of 25 *MiOFP*s in five tissues, including flower buds, leaves, stems, unripe, and ripe fruits (Figure 4). The findings indicated that the expression of MiOFP was primarily observed in flowers, unripe fruits, and stems, whereas its expression level in leaves and ripe fruits was generally limited. In addition, genes have different expression patterns in different tissues, suggesting that functional diversification was present in this subfamily. For example, in Class 2, *MiOFP*1 was highly expressed in stems and *MiOFP*5 was highly expressed in unripe fruit. Notably, *MiOFP*4, *MiOFP*5, *MiOFP*11, *MiOFP*15, *MiOFP*21, *MiOFP*22, *MiOFP*23, and *MiOFP*25 exhibited a high expression levels in both flowers and unripe fruits, suggesting a potential role of OFP in developing tissues, particularly during the early stages of fruit development, which may influence fruit shape formation.

### 3.4. Morphological Analysis of Fruit Development

**Figure 5:**
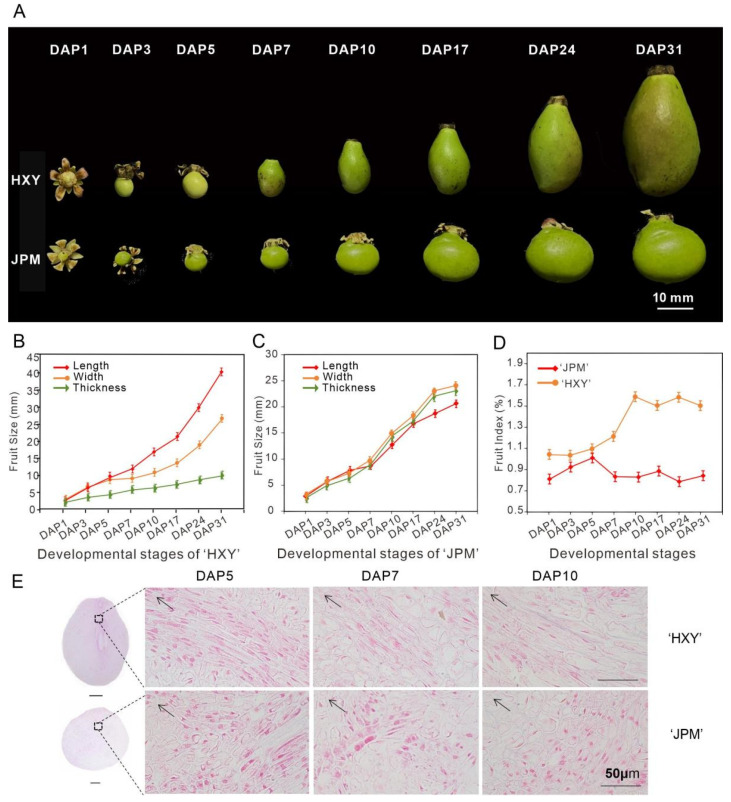
Morphological changes in the fruit shape of elongated and round mango throughout fruit development: (**A**) Fruit shapes at different developmental stages. Fruits of elongated mango ‘HXY’ and round mango ‘JPM’ are present in the top and bottom rows, respectively. DAP1, one day after pollination. Bar = 10 mm; (**B**) Fruit longitudinal length, transverse width, and lateral thickness of ‘HXY’; (**C**) Fruit longitudinal length, transverse width, and lateral thickness of ‘JPM’; (**D**) Fruit shape index of ‘JPM’ and ‘HXY’; (**E**) Paraffin sections of the fruit pulp were examined at developmental stages DAP5, DAP7, and DAP10. The black arrow represents the direction of the fruit stalk. The scale bar represents 50 μm. Sections were stained with hematoxylin–eosin staining (HE).

In order to investigate the mango fruit development, we recorded the fruit development process of two mango varieties, namely ‘Hongxiangya’ (Ivory-shaped) and ‘Jinpingmang’ (round), with a different fruit shape. The eight stages of the fruit development process were used to closely monitor the growth and development of the fruits within a month after pollination. The findings demonstrated a consistent augmentation in the longitudinal, transverse, and lateral diameters of the fruit starting from the day after pollination (referred to as DAP1, denoting the withering of sepals in the female flower, indicating a successful fruit set) and continuing until 31 days after pollination (referred to as DAP31, when the fruit shape index reaches a stable state) (Figure 5A). Substantial variations in morphological traits were observed between the two varieties starting from DAP5 and the greatest change in fruit shape index occurred at DAP7, the difference in fruit shape index reached its maximum value at DAP10. This observation indicates that the period encompassing DAP5, DAP7, and DAP10 is a critical stage in fruit development.

The longitudinal diameter of ‘HXY’ exhibited a faster rate of increase compared to the transverse and lateral diameters. In contrast, the growth rate of the longitudinal and lateral diameters of ‘JPM’ were similar, with the longitudinal diameter showing a slightly slower growth than the lateral diameter (Figure 5B,C). As a result, the fruit shape index of ‘HXY’ exhibited a rapid increase starting from DAP5, ultimately stabilizing at approximately 1.5 by DAP10. Conversely, the fruit shape index of ‘JPM’ decreased after DAP5 and consistently remained low (<1) thereafter (Figure 5D). This suggests that the variations in the fruit shape index of ‘HXY’ and ‘JPM’ occur during the critical period between DAP5 and DAP10, which is instrumental in determining the eventual development of elongated or round fruit types. 

Based on the analysis of these observations, it was obvious that DAP5–DAP10 was a critical period for the development of fruit shape variations. Previous research has suggested that cell division and expansion play a crucial role in regulating the shape and size of plant organs [33,34]. Specifically, the rate, duration, and orientation of cell division, as well as the uniform and non-uniform expansion of cells, greatly contribute to the final morphology of plant organs [35]. Therefore, paraffin sections were conducted on the fruits in these three stages to examine their cellular changes. The findings revealed that, in the ‘HXY’ fruits, the majority of cells underwent longitudinal elongation, whereas in the ‘JPM’ fruits, the cells experienced lateral elongation during the corresponding developmental stages (Figure 5E). This suggests that the differences in fruit shape between ‘HXY’ and ‘JPM’ are likely caused by differences in cell growth or the elongation direction during the early stage of fruit development (DAP5–DAP10). Simultaneously, the quantity of cells exhibiting longitudinal or transverse stretching at DAP5 was greater in the paraffin sections of the same size field, suggesting that the DAP5 stage might be a crucial phase for distinct cell division.

### 3.5. Expression Profile of MiOFP Genes in Two Varieties with Contrasting Fruit Shape

**Figure 6:**
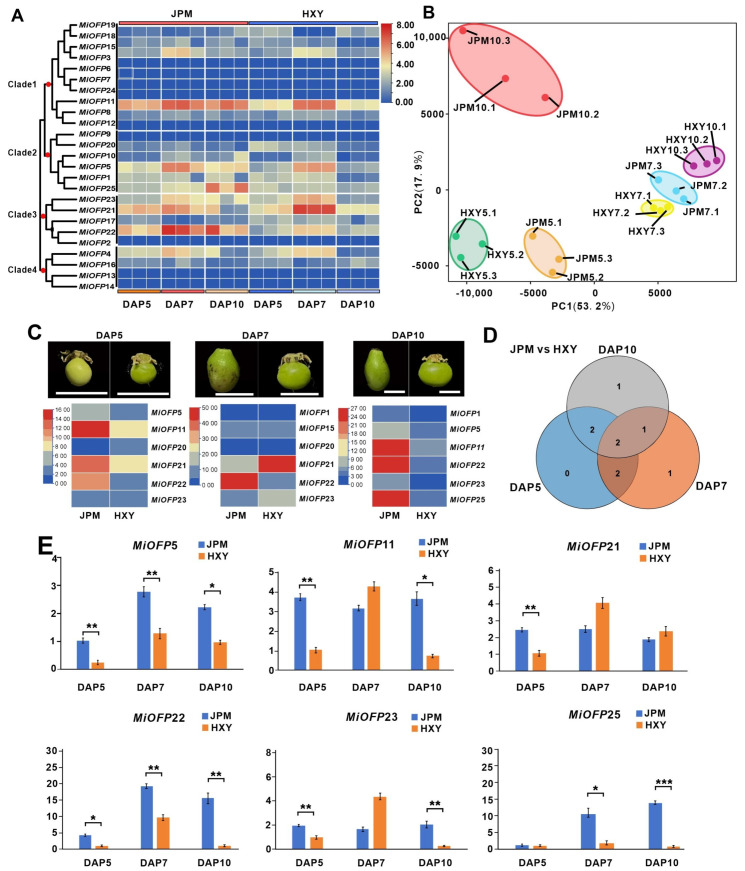
Expression pattern analysis of *MiOFP*s in mango: (**A**) Heat map of 25 *OFP* genes from three developmental stages in ‘HXY’ and ‘JPM’; (**B**) PCA of global gene expression levels of all 18 samples data; (**C**) Differentially expressed genes between elongated and round-shaped fruits at the DAP5, DAP7, and DAP10 stages; (**D**) Venn diagram showing number of differentially expressed MiOFP genes (log2FC > 2; padj < 0.05); (**E**) RT-qPCR validation of *MiOFP*5, *MiOFP*11, *MiOFP*15, *MiOFP*21, *MiOFP*22, *MiOFP*23, and *MiOFP*25 in the development of different mango fruit. Error bar represents standard deviation from three replicates. Different stars number above the bars represent significant differences (*p* < 0.05, LSD) among two varieties.* = *p* < 0.05, ** = *p* < 0.01, *** = *p* < 0.001.

To explore the character of *MiOFP*s in the regulation of the three pivotal fruit-shape development (DAP5, DAP7, and DAP10) mentioned by Figure 5E, transcriptome analysis was conducted on two mango cultivars (‘HXY’ and ‘JPM’), with ivory-shaped and round fruit, respectively (Figure 6A). Principal component analysis (PCA) was conducted to evaluate the correlation between biological replicates and the clustering patterns of the samples, and the results showed good reproducibility (Figure 6B). And, the pairwise comparison of the developing stages unmasked the common and exclusive differentially expressed transcripts at 5, 7, and 10 DAP between the two varieties. The analysis revealed that, among the 25 *MiOFP* genes, 6 *MiOFP* genes (*MiOFP*5, *MiOFP*11, *MiOFP*20, *MiOFP*21, *MiOFP*22, and *MiOFP*23) exhibited a differential expression at DAP5. Furthermore, six *MiOFP* genes (*MiOFP*1, *MiOFP*15, *MiOFP*20, *MiOFP*21, *MiOFP*22, and *MiOFP*23) displayed a differential expression at DAP7, and *MiOFP*1, *MiOFP*5, *MiOFP*11, *MiOFP*22, *MiOFP*23, and *MiOFP*25 were observed to have a differential expression at DAP10 in both varieties (Figure 6C). Furthermore, the RT-qPCR expression analysis of the six potential *MiOFP* genes mentioned above were performed between the two mango cultivars at the DAP5, DAP7, and DAP10 stages, and the results were observed to be consistent with the expression pattern of the transcriptome. Notably, *MiOFP5* and *MiOFP22* were consistently highly expressed in the round-shape fruit cultivar (‘JPM’) during all three stages (Figure 6C,E), which suggested its potential function in cell lateral elongation development, giving rise to the round fruit of mango. In addition, it was observed that *MiOFP*11, *MiOFP*21, and *MiOFP*23 exhibited notably high expression levels, specifically at DAP5 in JPM, and specifically at DAP7 in HXY, indicating their potential involvement in the early fruit development stage of cell elongation in JPM (Figure 6E).

## 4. Discussion

Known as the “King of Tropical Fruits”, mangoes are highly versatile and can be enjoyed fresh or utilized in various processed forms such as preserves, juice, and other related products. It is important to note that the shape of mangoes plays a critical role in determining the most suitable mechanical harvesting and processing methods. The *OFP* gene family has been found to be associated with fruit shape in various fruits. However, the genome-wide identification and comprehensive analysis of this gene family in mango have not been previously reported. This study revealed and comprehensively analyzed *OFP* family genes based on the whole-genome data for mango.

Recent studies showed that gene duplication played an important role not only in the process of genome rearrangement and expansion, but also in the diversity of gene function and the production of large numbers of gene families [36]. The number of *OFP* gene family members in different species was significantly different, with 19 *AtOFP*s in Arabidopsis, 31 *SlOFP*s in tomato, 35 *RsOFP*s in radish, 45 *ZmOFP*s in maize, and 33 *OsOFP*s in rice. Studies have already found that the average number of *OFP* genes was 19.91 in dicots, and 31.12 in monocots, indicating that the number of *OFP* genes in dicots was less than that of the monocots [22]. In this study, 25 *MiOFP* genes were systematically identified in mango’s Alphonso genome. The results of chromosomal locations analysis showed that 25 *MiOFP* genes were located on different chromosomes and might be the result of segmental duplication, and *MiOFP*3/4, *MiOFP*6/7, *MiOFP*13/14, and *MiOFP*15/16 were located in the similar position of the same chromosome, which might be the result of tandem duplication; *MiOFP*1, *MiOFP*2, *MiOFP*5, *MiOFP*8, *MiOFP*9, *MiOFP*10, *MiOFP*11, *MiOFP*12, *MiOFP*17, *MiOFP*18, *MiOFP*19, *MiOFP*20, *MiOFP*21, *MiOFP*22, *MiOFP*23, *MiOFP*24, and *MiOFP*25 were located on the different chromosomes and might be the result of segmental duplication. In addition, phylogenetic analysis divided the 25 MiOFPs into three main clades: Clades I, II, and III. Previous work showed that tomato *OVATE* and *SlOFP*1 genes that regulate pear-shaped tomato, *SlOFP*20 that controls tomato fruit shape, and *AtOFP*7/8 is considered as the candidate gene responsible for the shortening of Arabidopsis cotyledons [15], distributed in Clade Ⅱ, which suggests that the members in this clade including *MiOFP*1, *MiOFP*2, *MiOFP*5, *MiOFP*9, *MiOFP*10, *MiOFP*17, *MiOFP*20, *MiOFP*21, *MiOFP*22, *MiOFP*23, and *MiOFP*25 are likely involved in the regulation of the fruit shape and cotyledons elongation. 

The Ovate Family Proteins (OFP) family is a ubiquitously expressed transcription factor family in plants, playing diverse functions and roles [37]. The expression pattern analysis of *MiOFP* genes in different mango tissues revealed that the majority of them were expressed in the reproductive organs, which accordance with other plant [7,12]. In our study, three *MiOFP* genes (*MiOFP*6, *MiOFP*7, and *MiOFP*24) were undetectable in all tissues, suggesting a trend to degenerate these genes after gene duplication or the loss of gene functions during evolution. Notably, *MiOFP*4, *MiOFP*5, *MiOFP*11, *MiOFP*15, *MiOFP*21, *MiOFP*22, *MiOFP*23, and *MiOFP*25 exhibited high expression levels in both flowers and unripe fruits, suggesting a potential role of OFP in developing tissues, particularly during the early stages of fruit development, which may influence fruit shape formation (Figure 4). Previous studies have shown that *MiOFP*1 is highly expressed in the phloem tissue of the stem in childhood, potentially influencing plant height [38]. At *OFP*1, the homolog of *MiOFP*1 was reported to inhibit gibberellic acid (GA) biosynthesis by suppressing AtGA20ox1 activity, resulting in dwarf, rosette-like leaves [39]. In our study, *MiOFP*1 was specifically highly expressed in stems, which means that they may play a role in stem elongation. These findings suggest that OFP is implicated in the growth and development of diverse plant species, with potential variations in primary functions and expression patterns across different species. 

Fruit shape is a complex trait that results from a strict spatial and temporal control and coordination of overlapping and interconnected cellular events, cell division, and cell expansion, occurring with different onsets, rates, and duration [40]. In fruit trees, the members of the *OFP* gene family regulate the cell expansion and cell division of fruits, thereby affecting the appearance and shape of the fruits [5]. It has been reported that there were four periods of fruit development in mango, a period of initial limited growth (Stage 0: DAP0–4); a period of rapid increase (Stage I: DAP4–47); a period of delayed increase (Stage II: DAP47–53); and a second period of rapid increase and maturity (Stage III: DAP54–47). Size increases mainly occurred during Stage I, attaining 88% of the length at maturity [41]. In our observations of the fruit development process of “HXY” and “JPM”, DAP5–DAP10 is the key period for the change of fruit shape index, which determines the final development of the fruit shape elongated or round fruit type. Simultaneously, cytological analyses indicated a notable disparity in the orientation of cell elongation near the fruit stalk between HXY and JPM fruit at the onset of DAP5, suggesting a potential association between this discrepancy and the divergence in fruit shape formation.

Recent studies revealed that several *OFP*s negatively control the fruit length. For example, both *OVATE* and *SlOF*P20 regulate fruit length in tomato [7,16]. In the Wild Strawberry *Fragaria vesca*, *FvOFP1*, *FvOFP*11, *FvOFP*12, and *FvOFP*14 were highly expressed in achene and *CaOFP* genes may be involved in the formation of fruit type in pepper [42,43]. In our result, the differential expression of *MiOFP1*, *MiOFP*5, *MiOFP*11, *MiOFP1*5, *MiOFP*20, *MiOFP*21, *MiOFP*22, *MiOFP*23, and *MiOFP*25 were observed between ‘HXY’ and ‘JPM’ and the RT-qPCR analysis reveals the expression trends of *MiOFP*5, *MiOFP*11, *MiOFP*21, *MiOFP*22, *MiOFP*23, and *MiOFP*25 align with the expression trends observed in the transcriptome data. Furthermore, *MiOFP5* and *MiOFP22* was consistently highly expressed in round-shape fruit cultivar (‘JPM’) during all three stages (Figure 6C,E), which suggested its potential function in cell lateral elongation development, giving rise to round fruit of mango. As is known, *OVATE* gene was originally identified in tomato, and a natural mutation in OVATE causes the fruit to change from round to pear shape [7]. Considering the closer phylogenic relationship of *MiOFP*22 with the tomato *OVATE* gene, we believe the *MiOFP*22 is likely be a key gene involved in mango fruit shape formation.

## 5. Conclusions

The investigation identified 25 *OFP* genes in mango, with a predominant expression in reproductive organs. Morphological analysis indicated a divergence in fruit shape index at DAP5, with the most notable disparity observed at DAP10. Cytological observations revealed anisotropic growth in H‘XY’ and J‘PM’ cells post DAP5, with H‘XY’ cells exhibiting longitudinal growth and J‘PM’ cells showing lateral growth. The transcriptional analysis of fruit developmental stages unveiled temporal and spatial specificity in the expression patterns of various *MiOFP*. It is hypothesized that the differential *MiOFP* expression may be associated with the observed cellular anisotropic elongation, potentially contributing to the variation in fruit shape between H‘XY’ and J‘PM’. The present findings may lay the foundation for further studies to unravel the functions of mango OFP genes in fruit development and plant growth.

## Figures and Tables

**Table 1:** Primers sequences used in *MiOFP*s in this study.

Primer	Forward Primer (5′–3′)	Reverse Primer (5′–3′)	Amplicon Size
*MiOFP*5	TCCGAAAGTTGTGGTCATCGA	ACCACCACAAAGCTCTCCG	342
*MiOFP*11	TCGCCACCATTTTCGCCT	CCCGCCTCCTTTGAGTGG	227
*MiOFP*21	ACAGCACCACCGTTACCG	TCACCACCGCAAAGCTGT	251
*MiOFP*22	CCCTCCACTCTCCCGTCA	ATCGCGGAGGAAACGTGG	357
*MiOFP*23	GATGCCCAGAAGCCAGCA	CGATATGGGCGAGGCAGG	246
*MiOFP*25	TAGCTGCCGTGCCACAAT	AGAGTCACCGGCAGCAAC	391
*MiActin*	ATCTGCTGGAAGGTGCTGAG	CCAAGCAGCATGAAGATCAA	377

**Table 2:** Properties of *OFP*s identified from mango genome.

Gene Name	Gene ID	Chromosome Location	Size/aa	Molecular Weight/Da	pI	Hydro- Pathicity	Subcellular Localization
*MiOFP*1	LOC123225041	Chr01:1286519–1287906	392	44,485.46	9.55	−0.744	Chloroplast
*MiOFP*2	LOC123195071	Chr01:6284252–6285495	356	40,684.17	9.47	−0.658	Chloroplast
*MiOFP*3	LOC123193314	Chr01:21257006–21258100	280	30,946.09	4.99	−0.542	Nucleus
*MiOFP*4	LOC123192493	Chr01:21271282–21272056	186	20,802.65	9.66	−0.594	Chloroplast
*MiOFP*5	LOC123211132	Chr03:5224343–5225845	342	39,396.61	9.33	−0.82	Chloroplast
*MiOFP*6	LOC123210957	Chr03:7301842–7302579	245	28,217.85	6.96	−0.66	Chloroplast
*MiOFP*7	LOC123210960	Chr03:7306821–7311155	246	27,779.89	4.72	−0.496	Nucleus
*MiOFP*8	LOC123212208	Chr03:9478321–9479265	234	26,334.21	4.85	−0.468	Chloroplast
*MiOFP*9	LOC123219021	Chr06:1870688–1872202	411	47,439.15	9.41	−0.944	Chloroplast
*MiOFP*10	LOC123219408	Chr06:3989550–3990915	317	35,881.41	9.71	−0.874	Chloroplast
*MiOFP*11	LOC123221257	Chr07:18070119–18071499	227	25,157.93	5.46	−0.512	Nucleus
*MiOFP*12	LOC123223125	Chr08:13809269–13810321	234	26,385.33	5.68	−0.476	Chloroplast
*MiOFP*13	LOC123226435	Chr09:14142901–14143877	183	20,445.09	6.08	−0.652	Chloroplast
*MiOFP*14	LOC123226449	Chr09:14365554–14366530	183	20,445.09	6.08	−0.652	Chloroplast
*MiOFP*15	LOC123228213	Chr10:6211065–6212266	288	31,890.43	4.94	−0.453	Nucleus
*MiOFP*16	LOC123228223	Chr10:6224341–6225119	178	20,108.71	9.37	−0.626	Chloroplast
*MiOFP*17	LOC123230016	Chr11:2215612–2216944	318	36,541.54	9.54	−0.828	Mitochondrion
*MiOFP*18	LOC123192669	Chr12:5878219–5879439	239	26,570.41	4.62	−0.339	Nucleus
*MiOFP*19	LOC123193551	Chr12:11701597–11702637	235	26,093.09	4.92	−0.298	Nucleus
*MiOFP*20	LOC123192568	Chr12:14354392–14355878	394	45,728.22	9.52	−0.962	Chloroplast
*MiOFP*21	LOC123194383	Chr13:11784106–11785381	303	35,157.89	9.67	−0.795	Chloroplast
*MiOFP*22	LOC123199657	Chr16:1741505–1742791	357	40,800.53	9.43	−0.642	Chloroplast
*MiOFP*23	LOC123200363	Chr17:2891367–2892514	296	33,965.45	9.53	−0.758	Mitochondrion
*MiOFP*24	LOC123200256	Chr17:11932352–11933258	255	28,843.27	5.77	−0.64	Chloroplast
*MiOFP*25	LOC123202271	Chr18:2977275–2978794	391	44,206.97	9.56	−0.8	Chloroplast

## Data Availability

The RNA-seq data are obtained from NCBI (https://www.ncbi.nlm.nih.gov/Traces/study/ (accessed on 20 December 2023)), and the accession numbers are SRR27749143, SRR27749144, SRR27749145, SRR27749146, SRR27749147, SRR27749148, SRR27749149, SRR27749150, SRR27749151, SRR27749155, SRR27749153, SRR27749154, SRR27749155, SRR27749156, SRR27749157, SRR27749158, SRR27749159, and SRR27749160. Submitted data will remain private until related manuscript has been accepted. All data generated or analyzed are included within the article.

## References

[1] Zhang C., Xie D., Bai T., Luo X., Zhang F., Ni Z., Chen Y. (2020). Diversity of a Large Collection of Natural Populations of Mango (*Mangifera indica* Linn.) Revealed by Agro-Morphological and Quality Traits. Diversity.

[2] Kulkarni M.M., Burondkar M.M., Dalvi N.V., Salvi B.R., Haldankar P.M., Bhattacharyya T. (2019). Mango Fruit Size Diversity found in Konkan. Adv. Agric. Res. Technol. J..

[3] Zhu Q., Deng L., Chen J., Rodríguez G.R., Sun C., Chang Z., Yang T., Zhai H., Jiang H., Topcu Y. (2023). Redesigning the tomato fruit shape for mechanized production. Nat. Plants.

[4] van der Knaap E., Tanksley S.D. (2003). The making of a bell pepper-shaped tomato fruit: Identification of loci controlling fruit morphology in Yellow Stuffer tomato. Theor. Appl. Genet..

[5] Zhou H., Ma R.J., Gao L., Zhang J., Zhang A., Zhang X., Ren F., Zhang W., Liao L., Yang Q. (2021). A 1.7-Mb chromosomal inversion downstream of a *PpOFP1* gene is responsible for flat fruit shape in peach. Plant Biotechnol. J..

[6] Gao A., Chen Y., Luo R., Huang J., Zhao Z., Wang W., Wang Y., Dang Z. (2019). Development Status of Chinese Mango Industry in 2018. Adv. Agric. Hortic. Entomol..

[7] Liu J., Joyce V., Cong B., Tanksley S.D. (2002). A new class of regulatory genes underlying the cause of pear-shaped tomato fruit. Proc. Natl. Acad. Sci. USA.

[8] Gui B., Wang Y. (2007). Cloning and Sequence Analysis of ovate Orthologous Gene in Tobacco (*Nicotiana tabacum* L.). Plant Physiol. Commun..

[9] Rodríguez G.R., Muños S., Anderson C., Sim S.C., Michel A., Causse M., Gardener B.B.M., Francis D., van der Knaap E. (2011). Distribution of *SUN*, *OVATE*, *LC*, and *FAS* in the Tomato Germplasm and the Relationship to Fruit Shape Diversity. Plant Physiol..

[10] Tsaballa A., Pasentsis K., Darzentas N., Tsaftaris A.S. (2011). Multiple evidence for the role of an Ovate-like gene in determining fruit shape in pepper. BMC Plant Biol..

[11] Wang S., Chang Y., Guo J., Zeng Q., Ellis B., Chen J. (2011). Arabidopsis Ovate Family Proteins, a Novel Transcriptional Repressor Family, Control Multiple Aspects of Plant Growth and Development. PLoS ONE.

[12] Huang Z., Van H., Gonzalez G., Xiao H., van der Knaap E. (2013). Genome-wide identification, phylogeny and expression analysis of *SUN*, *OFP* and *YABBY* gene family in tomato. Mol. Genet. Genom..

[13] Wang S., Chang Y., Guo J., Chen J. (2007). Arabidopsis Ovate Family Protein 1 is a transcriptional repressor that suppresses cell elongation. Plant J..

[14] Schmitz A.J., Begcy K., Sarath G., Walia H. (2015). Rice Ovate Family Protein 2 (*OFP2*) alters hormonal homeostasis and vasculature development. Plant Sci..

[15] Yang C., Ma Y., He Y., Tian Z., Li J. (2018). *OsOFP*19 modulates plant architecture by integrating the cell division pattern and brassinosteroid signaling. Plant J..

[16] Wu S., Zhang B., Keyhaninejad N., Rodriguez G.R., Kim H.J., Chakrabarti M., Illa-Berenguer E., Taitano N.K., Gonzalo M.J., Díaz A. (2018). A common genetic mechanism underlies morphological diversity in fruits and other plant organs. Nat. Commun..

[17] Snouffer A., Kraus C., van der Knaap E. (2020). The shape of things to come: Ovate family proteins regulate plant organ shape. Curr. Opin. Plant Biol..

[18] Wang Y., Wang Q., Hao W., Sun H., Zhang L. (2020). Characterization of the *OFP* Gene Family and its Putative Involvement of Tuberous Root Shape in Radish. Int. J. Mol. Sci..

[19] Liu D., Sun W., Yuan Y., Zhang N., Hayward A., Liu Y., Wang Y. (2014). Phylogenetic analyses provide the first insights into the evolution of OVATE family proteins in land plants. Ann. Bot..

[20] Yu H., Jiang W., Liu Q., Zhang H., Piao M., Chen Z. (2015). Expression Pattern and Subcellular Localization of the Ovate Protein Family in Rice. PLoS ONE.

[21] Yuan Y., Zhang Y., Gao S., Tao J. (2016). Bioinformatics and Expression of the *OVATE* Gene Family in Grape. Sci. Agric. Sin..

[22] Qi W., Luo Z., Li H., Zhang Z., Yan C., Wang J., Liu M. (2021). Identification and Bioinformatics Analysis of OVATE Gene Family in Chinese Jujube. Mol. Plant Breed..

[23] Li H., Dong Q., Zhao Q., Ran K. (2019). Genome-wide identification, expression profiling, and protein-protein interaction properties of ovate family proteins in apple. Tree Genet. Genomes.

[24] Wang P., Luo Y., Huang J., Gao S., Zhu G., Dang Z., Gai J., Yang M., Zhu M., Zhang H. (2020). The genome evolution and domestication of tropical fruit mango. Genome Biol..

[25] Chen C., Wu Y., Li J., Wang X., Zeng Z., Xu J., Liu Y., Feng J., Chen H., He Y. (2023). TBtools-II: A “one for all, all for one” bioinformatics platform for biological big-data mining. Mol. Plant.

[26] Katoh K., Standley D.M. (2013). MAFFT Multiple Sequence Alignment Software Version 7: Improvements in Performance and Usability. Mol. Biol. Evol..

[27] Nguyen L.T., Schmidt H.A., von Haeseler A., Minh B.Q. (2015). IQ-TREE: A Fast and Effective Stochastic Algorithm for Estimating Maximum-Likelihood Phylogenies. Mol. Biol. Evol..

[28] Kalyaanamoorthy S., Minh B.Q., Wong T.K.F., von Haeseler A., Jermiin L.S. (2017). ModelFinder: Fast model selection for accurate phylogenetic estimates. Nat. Methods.

[29] Ada T.F., Delia W. (2014). Tissue Processing and Hematoxylin and Eosin Staining. Histopathol. Methods Protoc. Methods Mol. Biol..

[30] Chen S., Zhou Y., Chen Y., Gu J. (2018). fastp: An ultra-fast all-in-one FASTQ preprocessor. Bioinformatics.

[31] Wagner G.P., Kin K., Lynch V.J. (2012). Measurement of mRNA abundance using RNA-seq data: RPKM measure is inconsistent among samples. Theory Biosci..

[32] Livak K.J., Schmittgen T.D. (2001). Analysis of relative gene expression data using real-time quantitative PCR and the 22^−ΔΔCt^ Method. Methods.

[33] Gao H., Li P., Donf T. (2016). Advances in the Study off Actors Influencing Fruit Shape. J. Trop. Biol..

[34] Guo J., Cao K., Li Y., Yao J., Deng C., Wang Q., Zhu G., Fang W., Chen C., Wang X. (2018). Comparative Transcriptome and Microscopy Analyses Provide Insights into Flat Shape Formation in Peach (*Prunus persica*). Front. Plant Sci..

[35] Gillaspy G., Hilla B.D., Wilhelm G. (1993). Fruits: A Developmental Perspective. Plant Cell.

[36] Cannon S.B., Mitra A., Baumgarten A., Young N.D., May G. (2004). The roles of segmental and tandem gene duplication in the evolution of large gene families in *Arabidopsis thaliana*. BMC Plant Biol..

[37] Wang S., Chang Y., Ellis B. (2016). Overview of OVATE FAMILY PROTEINS, A Novel Class of Plant-Specific Growth Regulators. Front. Plant Sci..

[38] Zhang Y.L., Zhu J.W., Lao A., Li Y.Z., Xia L.M., MO X., Hu W.L., He X.H., Luo C. (2023). Expression and functional analysis of MiOFP1 gene in mango. J. Fruit Sci..

[39] Bleckmann A., Weidtkamp-Peters S., Seidel C.A.M., Simon R. (2010). Stem Cell Signaling in Arabidopsis Requires CRN to Localize CLV2 to the Plasma Membrane. Plant Physiol..

[40] Mauxion J.P., Chevalier C., Gonzalez N. (2021). Complex cellular and molecular events determining fruit size. Trends Plant Sci..

[41] Chen H.B., Huang M.Y. (1995). Fruit growth and abscission of the mango (*Mangifera indica* L.) ZiHua. J. South China Agric. Univ..

[42] Xu X., Wang X., Zhou S., Huang X., Liu P., Ma B., Chen X. (2024). Genome-Wide Identification and Characterization of the OFP Gene Family in the Wild Strawberry *Fragaria vesca*. Agronomy.

[43] Luo Y., Yang S., Luo X., Li J., Li T., Tang X., Liu F., Zou X., Qin C. (2022). Genomewide analysis of OFP gene family in pepper (*Capsicum annuum* L.). Front. Genet..

